# Bacterial–Fungal Interactions in the Kelp Endomicrobiota Drive Autoinducer-2 Quorum Sensing

**DOI:** 10.3389/fmicb.2019.01693

**Published:** 2019-07-31

**Authors:** Anne Tourneroche, Raphaël Lami, Cédric Hubas, Elodie Blanchet, Marine Vallet, Karine Escoubeyrou, Alain Paris, Soizic Prado

**Affiliations:** ^1^Unité Molécules de Communication et Adaptation des Microorganismes (MCAM), Muséum National d’Histoire Naturelle (MNHN), Centre National de la Recherche Scientifique (CNRS), CP 54, Paris, France; ^2^CNRS, Laboratoire de Biodiversité et Biotechnologies Microbiennes (LBBM), USR3579, Observatoire Océanologique de Banyuls, Sorbonne Université, Banyuls-sur-Mer, France; ^3^Muséum National d’Histoire Naturelle, UMR BOREA 7208 MNHN-Sorbonne Université-CNRS-UCN-UA-IRD, Station Marine de Concarneau, Paris, France; ^4^CNRS, Observatoire Océanologique de Banyuls, Sorbonne Université, FR3724, Banyuls-sur-Mer, France

**Keywords:** quorum sensing (QS), AI-2, bacterial–fungal interaction, kelp microbiota, algal holobiont

## Abstract

Brown macroalgae are an essential component of temperate coastal ecosystems and a growing economic sector. They harbor diverse microbial communities that regulate algal development and health. This algal holobiont is dynamic and achieves equilibrium *via* a complex network of microbial and host interactions. We now report that bacterial and fungal endophytes associated with four brown algae (*Ascophyllum nodosum*, *Pelvetia canaliculata*, *Laminaria digitata*, and *Saccharina latissima)* produce metabolites that interfere with bacterial autoinducer-2 quorum sensing, a signaling system implicated in virulence and host colonization. Additionally, we performed co-culture experiments combined to a metabolomic approach and demonstrated that microbial interactions influence production of metabolites, including metabolites involved in quorum sensing. Collectively, the data highlight autoinducer-2 quorum sensing as a key metabolite in the complex network of interactions within the algal holobiont.

## Introduction

Large marine brown algae, i.e., kelp, are an essential component of temperate coastal ecosystems. Indeed, these organisms are important primary producers that generate a specific habitat and thereby shape coastal marine life (Egan et al., 2013). In addition, kelp farming has been a growing economic sector over the last decades (FAO, 2018).

Like most eukaryotes, macroalgae are colonized by various microorganisms (the microbiota) that interact with them throughout the life cycle, and that modify their physiology (Wahl et al., 2012; Egan et al., 2013; Singh and Reddy, 2014). For example, commensal bacteria have profound effects on seaweed development, nutrition, and defense (Wahl et al., 2012; Singh and Reddy, 2014; Tapia et al., 2016). Algal tissues are also asymptomatically colonized by filamentous fungi (Debbab et al., 2012), although these fungi and their role are yet to be fully characterized (Fries, 1979; Zuccaro et al., 2003, 2008; Loque et al., 2010; Jones et al., 2012). Previously, we isolated and characterized the molecular diversity of cultivable fungi in different parts of the brown algae *Ascophyllum nodosum*, *Pelvetia canaliculata*, *Laminaria digitata*, and *Saccharina latissima.* We also found that metabolites produced by endophytic fungi are key mediators of interactions among macroalgae, their fungal microbiota, and protistan pathogens (Vallet et al., 2018).

Endophytic bacteria are likely to interact with fungi in the algal host to maintain the host-microbiota equilibrium and thus contribute to host health (Deveau et al., 2018; Hassani et al., 2018). Indeed, structural changes in the microbiota, i.e., dysbiosis, have been linked to disease in marine organisms and seaweeds (Fernandes et al., 2012; Zozaya-Valdes et al., 2015; Egan and Gardiner, 2016). However, the mechanisms underlying bacterial–fungal homeostasis remain unclear, although they appear crucial to macroalgal physiology not only in nature but also in farms, highlighting their importance in light of intensifying algal culture (Gachon et al., 2010).

Bacterial–fungal interactions consist of multiple and concomitant mechanisms ranging from nutrient competition to antibiosis, many of which depend on chemical signaling. Quorum sensing, which allows bacteria to coordinate gene expression based on the density of specific signaling molecules, is of particular interest, since it is essential for virulence, colonization, biofilm formation, and toxin production (Atkinson and Williams, 2009). Indeed, various types of quorum signals, also known as auto-inducers (AI), are already known. Some have been found in only one genus whereas others, such as type 1 (AI-1) or type 2 (AI-2), are present in various bacterial genera. Indeed, AI-2 molecules appear widespread among prokaryotes, and is produced by over 50% of sequenced bacterial species, including both Gram-positive and Gram-negative species (Hammer and Bassler, 2003; Federle, 2009). AI-2 molecules are derived from a common precursor, (*S*)-4,5-dihydroxypentane-2,3-dione (DPD), which is synthesized by the enzyme LuxS. By spontaneous cyclization, this precursor is transformed to 4-hydroxy-5-methyl-3(*2H*)furanone, (*2R*,*4S*)-2-methyl-2,3,3,4-tetrahydroxytetrahydrofuran, and, especially in marine environments, to the furanosyl borate diester (Hardie and Heurlier, 2008).

Various marine bacteria were found to produce AI-2 (Bodor et al., 2008; Doberva et al., 2015; Pérez-Rodríguez et al., 2015), although it is considered in some species to be a metabolic by product and not a signaling molecule (Rezzonico and Duffy, 2008). Nevertheless, many studies showed that AI-2 regulates niche-specific behaviors in commensal and pathogenic bacteria, including biofilm formation or dispersion, cell division, virulence, bioluminescence, and motility (Hammer and Bassler, 2003; Hardie and Heurlier, 2008; Federle, 2009). Accordingly, secreted AI-2 is now recognized as a key signaling molecule that affects bacterial behavior at species and community level (Whiteley et al., 2017).

Strikingly, quorum sensing molecules are not exclusively produced by bacteria. Indeed, these compounds were also shown to regulate fungal morphogenesis, germination, apoptosis, biofilm development, or pathogenicity (Wongsuk et al., 2016). Importantly, some recent studies showed that both bacterial and fungal quorum sensing compounds mediate cross-kingdom signaling. For instance, eukaryotes may interfere with bacterial quorum sensing (Martín-Rodríguez et al., 2014; Ismail et al., 2016), while bacteria may react to fungal quorum signals (Cugini et al., 2007; Fourie et al., 2016). Cross-kingdom signaling is also modulated by quorum sensing inhibitors, i.e., quorum quenchers (Grandclément et al., 2016; Rolland et al., 2016). To date, halogenated furanones synthesized by the marine red algae *Delisea pulchra* are the best-studied naturally occurring quorum sensing inhibitors in eukaryotes. These compounds regulate bacterial colonization of algal surfaces by interfering with AI-1 and AI-2 (Defoirdt et al., 2007; Harder et al., 2012).

Despite these breakthroughs, the role of AI-2 quorum sensing in marine environments in general, and in holobionts in particular, remains poorly characterized (Doberva et al., 2015; Hmelo, 2017). One important shortcoming is the lack of quantitative measurements, since AI-2 itself is difficult to quantify (Wang et al., 2018). In this study, we hypothesized that AI-2 quorum sensing is involved in interspecies chemical signaling among endophytic fungi and bacteria in seaweeds. Accordingly, we first isolated and molecularly characterized the cultivable bacteria associated with the brown algae *A. nodosum*, *P. canaliculata*, *L. digitata*, and *S. latissima*. These bacteria were then investigated for their ability to produce or inhibit production of AI-2 along with fungi previously isolated from the same samples (Vallet et al., 2018). Co-cultures experiments, between several fungal and bacterial endophytic strains isolated from *S. latissima* microbiota combined with metabolomics approach pointed out that inter-species interactions involve metabolites production that modulates AI-2 production. Altogether these results suggest that dynamic interactions driven by microbial metabolites may occur within the microbiota and impact AI-2 QS signaling.

## Materials and Methods

### Sampling and Endophyte Isolation

Fungi and bacteria were previously isolated from the brown algae *L. digitata*, *S. latissima*, *A. nodosum*, and *P. canaliculata*. *L. digitata*, and *A. nodosum* were collected in triplicate in Roscoff, France, in January 2013. Samples of all four species were also collected in triplicate in Oban, Scotland, in July 2013. Algae were surface-sterilized with 70% ethanol and 0.1% sodium hypochlorite, and cut into small pieces. Around 4,600 of these pieces were then aseptically transferred to different solid media, using at least 10 replicates from each algal part on each type of medium (Supplementary Table S1). Resulting cultures were then grown and preserved following previously described protocols (Vallet et al., 2018).

### Taxonomic Identification of Endophytic Bacteria

Genomic DNA was extracted with Wizard^®^ Genomic DNA Purification Kit (Promega, Charbonnières-les-Bains, France) from single colonies of 209 bacterial isolates that were grown in marine broth. 16S rRNA genes were then amplified using 2× KAPA2G Ready Mix (Clinisciences, Nanterre, France), 1 μL bacterial DNA, and the universal primers 27F mod (5′-AGRGTTTGATCMTGGCTCAG-3′) and 1492R mod (5′-TACGGYTACCTTGTTAYGACTT-3′). Targets were amplified over one cycle of denaturation at 94°C for 5 min, 35 cycles at 94°C for 15 s, 50°C for 15 s, and 72°C for 20 s, and final extension at 72°C for 10 min. PCR products were sequenced by Sanger sequencing on the Bio2Mar platform (Observatoire Océanologique, Banyuls-sur-Mer, France), using primer 907R (Eurofins MWG Operon, Ebersberg, Germany).

The quality of each sequence was checked manually and the closest match in NCBI databases was determined by BLAST (Altschul et al., 1990). Further, sequences were aligned in Muscle, as implemented in MEGA 7.0 (Edgar, 2004; Kumar et al., 2016). Alignments were reviewed manually to verify mismatches, and a phylogenetic tree was constructed by maximum likelihood using the K2, G+I model. The reliability of each node in the tree was assessed by bootstrapping over 500 replicates.

### Screening for the Production of Quorum Sensing Mediators

The QS bioluminescent reporter strain *Vibrio campbellii* MM32 (***luxN***::Cm, ***luxS***::Tn5Kan) was used to detect AI-2 in bacterial and fungal extracts, as previously described (Miller et al., 2004). The receptor *luxN* is mutated in this strain to abolish sensing of acyl homoserine lactones, while the synthase gene *luxS* is mutated to abolish AI-2 production but not sensing. It was previously constructed by introducing *luxS*::Tn5Kan onto the chromosome of strain JAF305 (*luxN*::Cm) (Bassler et al., 1993; Freeman and Bassler, 1999).

To obtain bacterial supernatants, 1 mL was collected from each of 209 bacterial cultures grown for 24 h at 22°C in marine broth. Samples were then centrifuged at 17,000 *× g* for 10 min, and resulting supernatants were filtered at 0.22 μm. To obtain fungal extracts, 43 fungal isolates were grown for 3 weeks at 19°C in MEA/ASW medium, and extracted three times with ethyl acetate. A detailed recipe of the medium is provided in Supplementary Table S1. Extracts were tested at a final concentration of 250 μg/mL, with final concentration of DMSO 2.5%.

Bacterial supernatants and fungal extracts were tested for the production of molecules interfering with AI-2 quorum sensing. Briefly, 20 μL of test samples and corresponding controls were mixed with 180 μL of *V. campbellii* MM32 diluted 1:5,000 and incubated at 30°C and 100 rpm. Luminescence and cell density (OD_620_) were measured after 24 h. Data were collected in triplicate, and luminescence change was calculated as (lumi_SN/E_ – lumi_Control_)/lumi_Control_, where lumi_SN/E_ is bioluminescence (normalized to cell density) from the reporter strain in the presence of supernatant or extract, and lumi_Control_ is bioluminescence (normalized to cell density) in the presence of either marine broth (when supernatants are tested) or DMSO (when extracts are tested).

### Quantification of AI-2 Precursor by LC–MS/MS

Due to low ionization potential and instability, AI-2 and DPD are not directly detectable by mass spectrometry (MS). However, quinoxaline derivatives of DPD, obtained by reaction with 4,5-dimethyl-1,2-phenylenediamine, are detectable by LC–MS/MS. DPD was thus quantified in bacterial supernatants after performing a derivatization reaction as described (Xu et al., 2017). Briefly, triplicate DPD standard solutions with concentration 2.6 nM–26 μM were obtained by diluting a stock solution of DPD (16.64 nM) in marine broth. To obtain quinoxaline derivatives, 250 μL of standard solution or supernatant was reacted with 250 μL of 0.1 mg/mL 4,5-dimethyl-1,2-phenylenediamine (Sigma, St. Louis, MO, United States) in 0.1M HCl. Samples were thoroughly mixed for 1 min, and incubated for 5 h at 25°C with agitation. Samples were then desalted with two volumes of water using Sep-Pak C18 SPE cartridges (Waters, Beverly, MA, United States), and eluted with two volumes of acetonitrile. Subsequently, samples were analyzed by LC–MS/MS on a Dionex Ultimate 3000 HPLC system coupled to a Q Exactive^TM^ Focus mass spectrometer (Thermo Fisher Scientific, Waltham, MA, United States) and fitted with an electrospray ionization source and a Hypersil GOLD C18 column (2.1 mm × 150 mm, 1.9 μm particle size; Thermo Scientific, Waltham, MA, United States) operating at 20°C. In this system, eluates are introduced directly into the mass spectrometer. LC–MS parameters are detailed in Supplementary Table S1. Data were collected using Xcalibur, in parallel reaction monitoring mode targeting the precursor ion at *m/z* 233.1285. The product ion at *m/z* 186.1140 was used for quantification.

### AI-2 Antagonist Activity in Fungal Extracts

To test AI-2 antagonist activity in fungal extracts, 20 μL samples were reacted as described with 180 μL of *V. campbellii* MM32 diluted 1:5,000 in marine broth and supplemented with 2 μM DPD (purchased from Rita Ventura’s research group at ITQB, Oeiras, Portugal). To confirm that loss of luminescence, if any, was not due to cytotoxicity, 100 μL of culture was reacted with 30 μL of 0.01% resazurin (Graça et al., 2013) immediately after measurement of luminescence, and fluorescence (λ_ex_: 530 nm, λ_em_: 590 nm) was measured after incubating for 4 h at 30°C with agitation. As control, a 96-well plate containing 20 μL of fungal extract in marine broth was assayed in the same manner to assess background luminescence, fluorescence, and absorbance.

### Co-culture Experiment: Culture Conditions and Impact on Quorum Sensing

Fungal and bacterial isolates were co-cultured in triplicate in marine broth supplemented with 10% malt extract, 4% glucose, and 1.5% agar, adjusted to pH 7, and plated. Plates were inoculated with 2 mL of a mixture of 2 × 10^4^ fungal spores and bacteria diluted to OD 0.1. Corresponding monocultures were prepared in triplicate in the same manner. Cultures were then incubated for 21 days at 19°C and on a 12-h light/dark cycle. Petri dishes containing only culture medium (*n* = 3) were used as blank. Cultures and corresponding controls were extracted with ethyl acetate for 30 min, in a sonicator at room temperature. Samples were then filtered through a filter paper, and dried under vacuum using a centrifugal evaporator. Extracts were tested for their impact on quorum sensing at a final concentration of 250 μg/mL, with final concentration of DMSO 2.5%. Briefly, 20 μL of extract were mixed with 180 μL of *V. campbellii* MM32 diluted 1:5,000 and incubated at 30°C and 100 rpm. Luminescence and cell density (OD_620_) were measured after 24 h. Data were collected in triplicate, and luminescence change was calculated as (lumi_E_ – lumi_Control_)/lumi_Control_, where lumi_E_ is bioluminescence (normalized to cell density) from the reporter strain in the presence of extract, and lumi_Control_ is bioluminescence (normalized to cell density) in the presence of DMSO. To assess the viability of the biosensor, 100 μL of culture was reacted with 30 μL of 0.01% resazurin (Graça et al., 2013) immediately after measurement of luminescence, and fluorescence (λ_ex_: 530 nm, λ_em_: 590 nm) was measured after incubating for 4 h at 30°C with agitation.

### Co-culture Experiment: LC–MS-Based Metabolomic Analysis

Dried extracts were solubilized in methanol at 0.5 mg/mL, and analyzed by HPLC–MS in one batch and in a random sequence. Samples were loaded onto a Dionex Ultimate 3000 HPLC system fitted with a C18 Acclaim^TM^ RSLC PolarAdvantage II column (2.1 mm × 100 mm, 2.2 μm pore size; Thermo Scientific, Waltham, MA, United States) operating at 40°C, and coupled to a Maxis II^TM^ QTOF mass spectrometer (Bruker, Bellerica, MA, United States) with an electrospray ionization source. Data were acquired with Data Analysis software. LC–MS parameters are listed in Supplementary Table S1. Raw LC–MS data were calibrated and converted to netCDF format using Data Analysis software (Bruker), and processed using the R package XCMS (Smith et al., 2006). Based on analytical conditions and raw data characteristics, final peak picking parameters were method = ‘centWave,’ ppm = 10, and peak width = c(5,20), while final grouping parameters were bw = 5, mzwid = 0.015, and retention time correction method = ‘obiwarp.’ Other parameters were set to default values. To limit noise from compounds already present in culture media, the dataset was filtered with an in-house script to retain only those features with intensity in at least one sample more than fivefold its average intensity in blank samples.

### Statistical Procedures

All analyses and graphs were performed using the R statistical framework (R Core Team, 2019). Van der Waerden tests followed by a *post hoc* test using the Fischer’s Least Significant Difference (LSD) criterion was performed to test the contrasting effect of mono and co-culture on QS activity. Multivariate analyses were done using the R library mixOmics (Rohart et al., 2017).

## Results

### Diversity of Cultivable Endophytic Bacteria From Brown Algae

**FIGURE 1:**
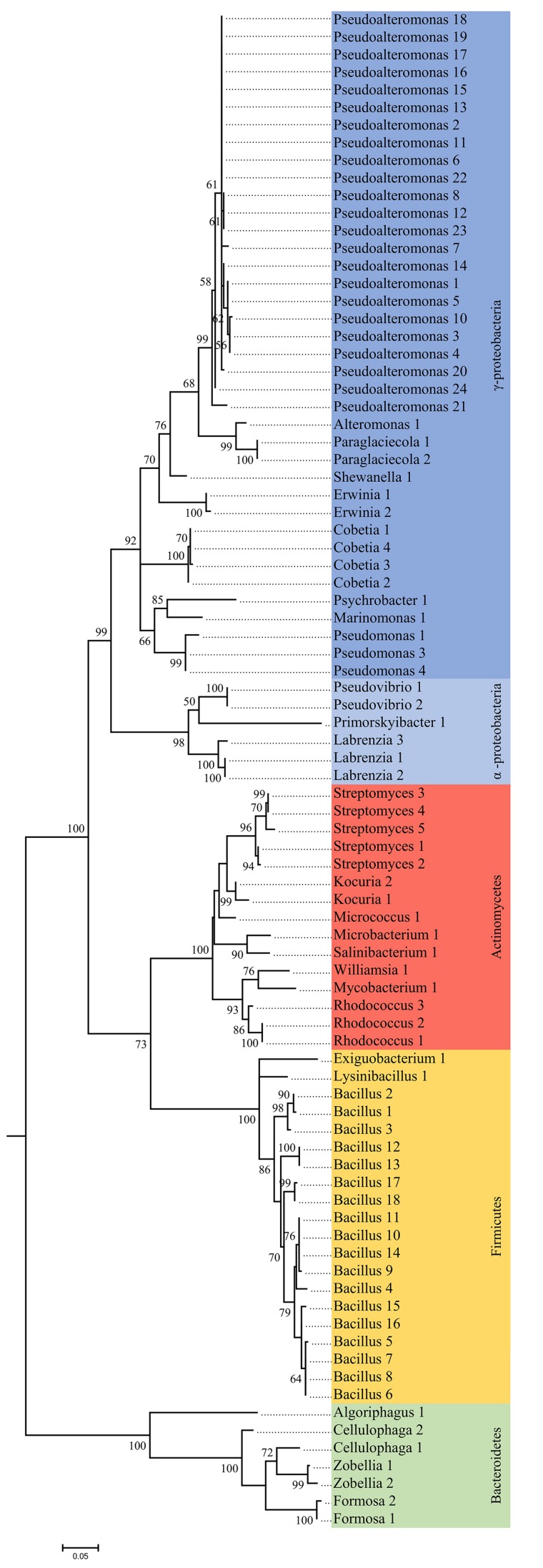
Phylogenetic tree of unique 16S rRNA sequences from bacteria isolated from four algal species. The tree was constructed by maximum likelihood using the K2, G+I model. The reliability of each node was assessed by bootstrapping over 500 replicates.

**FIGURE 2:**
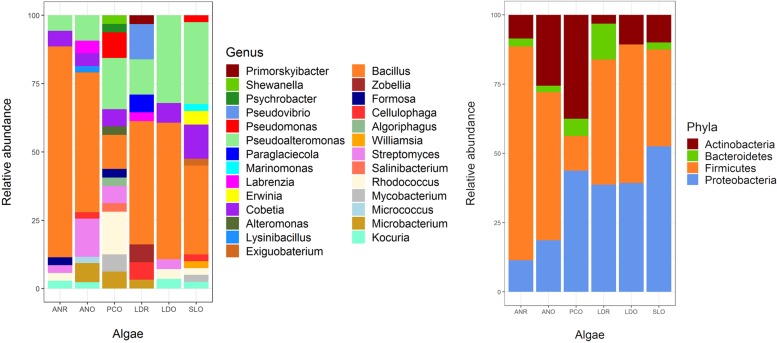
Abundance of cultivable bacterial genus (left) and phyla (right) by algal host and sampling site. ANR, *Ascophylum nodosum* from Roscoff; ANO, *A. nodosum* from Oban; PCO, *Pelvetia canaliculata* from Oban; LDR, *Laminaria digitata* from Roscoff; LDO, *L. digitata* from Oban; SLO, *Saccharina latissima* from Oban.

A total of 209 bacterial isolates was obtained, and classified based according to 16S rRNA genes into 4 phyla, 12 orders, 19 families, 27 genera, and 88 taxonomically unique units (Figures 1, 2 and Supplementary Table S2). The most abundant phyla in *L. digitata* and *S. latissima* were *Firmicutes* (comprising 47 and 35% of all isolates, respectively) and *Proteobacteria* (39 and 53%). In *P. canaliculata*, *Proteobacteria*, and *Actinobacteria* were predominant (44 and 38%), whereas *Firmicutes* accounted for 77% of bacteria isolated from *A. nodosum*. *Gammaproteobacteria* was between 50 and 100% of all *Proteobacteria* depending on algal species, although *Alphaproteobacteria* was occasionally present. On the other hand, *Bacillus* and *Pseudoalteromonas* were the most abundant genera in *S. latissima* (33 and 30%) and *L. digitata* (47 and 22%). Isolates from *P. canaliculata* were mostly *Pseudoalteromonas*, *Rhodococcus*, and *Bacillus* (19, 16, and 13%), whereas *Bacillus* was dominant in *A. nodosum* (63%). *Rhodococcus*, *Bacillus*, *Cobetia*, and *Pseudoalteromonas* were isolated from all four algal species.

### Production of AI-2 Compounds by Bacterial Endophytes

Most (86%) bacterial endophytes isolated from brown algae elicited an increase in luminescence from *V. campbellii* MM32, a quorum sensing reporter strain (Table 1 and Supplementary Data S1). Strikingly, 10% of isolates boosted luminescence by over 50%, including a *Kocuria* isolate, six *Bacillus* isolates, and 13 *Proteobacteria*. Indeed, a *Marinomonas* isolate and five *Cobetia* isolates increased luminescence by at least 100%. In contrast, all but one *Pseudoalteromonas* isolate increased luminescence by less than 50%.

**TABLE 1:** Induction of luminescence in the biosensor *V. campbellii* MM32 by bacterial supernatants.

**Genus**	**No induction**	**Induction inferior to 50%**	**Induction between 50 and 100%**	**Induction superior or equal to 100%**
*Kocuria*	1	2	1	0
*Microbacterium*	1	5	0	0
*Micrococcus*	0	1	0	0
*Mycobacterium*	1	2	0	0
*Rhodococcus*	3	5	0	0
*Salinibacterium*	0	1	0	0
*Streptomyces*	3	7	0	0
*Williamsia*	0	1	0	0
*Algoriphagus*	1	0	0	0
*Cellulophaga*	2	2	0	0
*Formosa*	0	2	0	0
*Zobellia*	2	0	0	0
*Bacillus*	1	88	6	0
*Exiguobacterium*	0	1	0	0
*Lysinibacillus*	0	1	0	0
*Alteromonas*	0	1	0	0
*Cobetia*	0	2	5	5
*Erwinia*	0	1	1	0
*Labrenzia*	1	2	0	0
*Marinomonas*	0	0	0	1
*Paraglaciecola*	2	0	0	0
*Pseudoalteromonas*	9	27	1	0
*Pseudomonas*	0	4	0	0
*Pseudovibrio*	3	1	0	0
*Psychrobacter*	0	1	0	0
*Shewanella*	0	1	0	0
*Primorskyibacter*	0	1	0	0

*Numbers are bacterial isolates per genus.*

**FIGURE 3:**
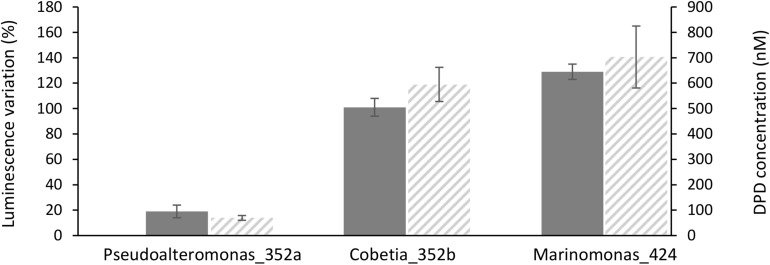
Induction of luminescence in the biosensor *Vibrio campbellii* MM32 by (gray bars), and DPD concentration in three bacterial supernatants (hatched bars). Luminescence induction was quantified as described in Section “Materials and Methods.” Error bars indicate standard deviation.

To confirm these results, DPD production by *Marinomonas* 424 and *Cobetia* 352b, two of the strongest inducers of luminescence, was quantified by LC–MS/MS. For comparison, DPD production was also quantified in *Pseudoalteromonas* 352a, which was co-isolated with *Cobetia* 352b and is a weak inducer of luminescence. Interestingly, culture supernatants from *Marinomonas* 424 and *Cobetia* 352b contained similar levels of DPD (692 and 585 nM, respectively), whereas a lower amount was found in the supernatant from *Pseudoalteromonas* 352a (66 nM). Thus, our results based on LC–MS/MS confirmed the quorum sensing patterns observed using the biosensor-based approach (Figure 3).

### Impact of Metabolites of Endophytic Fungi on Quorum Sensing

**FIGURE 4:**
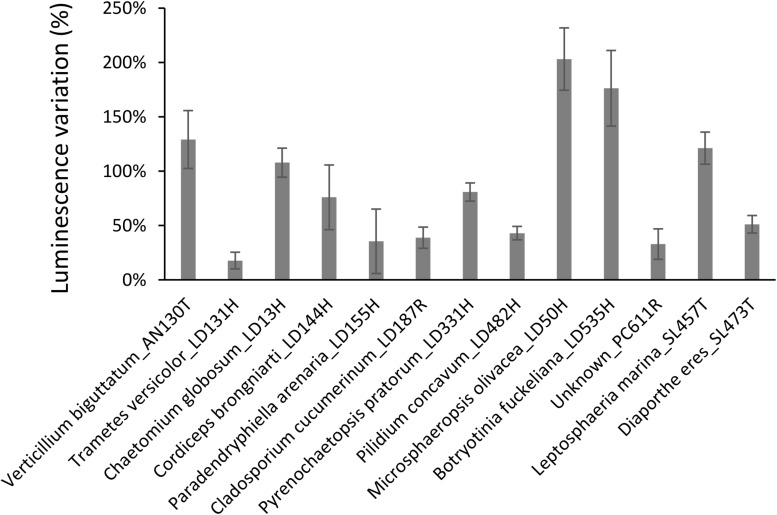
Induction of luminescence in the biosensor *V. campbellii* MM32 by fungal extracts. Error bars indicate standard deviation.

**FIGURE 5:**
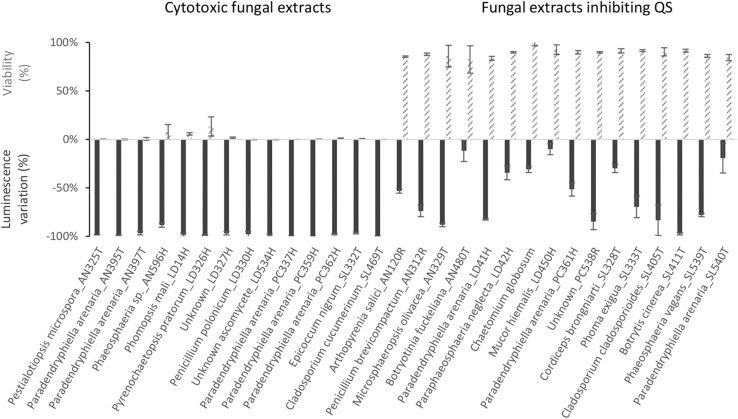
Impact of fungal extracts on quorum sensing in and viability of the biosensor *V. campbellii* MM32, as measured, respectively, by inhibition of luminescence in the presence of 2 μM DPD and by resazurin test. Assays are described in Section “Materials and Methods.” Error bars indicate standard deviation.

As presented in Figure 4, extracts from 13 fungi boosted the luminescence from the biosensor. Of these, eight (*Verticillium biguttatum* AN130T, *Chaetomium globosum* LD13H, *Microsphaeropsis olivacea* LD50H, *Botryotinia fuckeliana* LD535H, *Leptosphaeria marina* SL457T, and *Diaporthe eres* SL473T) increased the luminescence by over 50%. Conversely, extracts from 30 fungi strongly diminished the luminescence, as shown in Figure 5. For 14 of these extracts, the loss of luminescence was due to toxicity against the biosensor *V. campbellii* MM32. In contrast, the other 16 extracts evidently inhibited AI-2, blocking the effects of 2 μM DPD but with very limited impact on the biosensor *V. campbellii* MM32 metabolism (Figure 5).

### Impact of Bacterial–Fungal Interactions on Quorum Sensing

**FIGURE 6:**
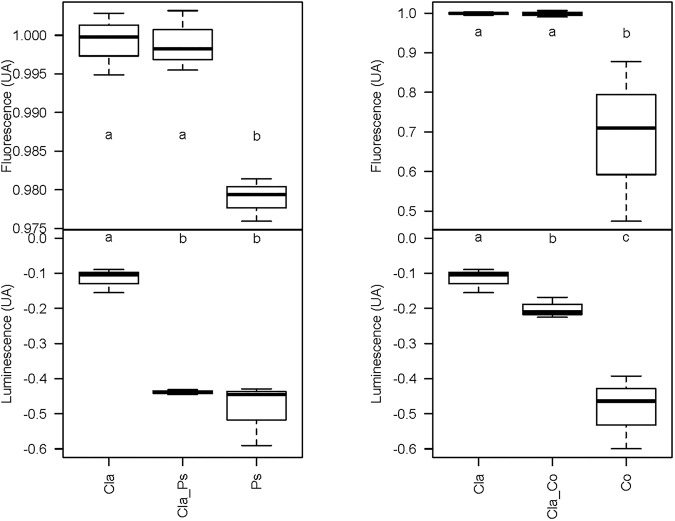
Box plot representing the variation of the luminescence and viability (fluorescence) of the biosensor *V. campbellii* MM32 by the bacterial or the fungal monoculture and the co-culture. Cla, Cladosporium monocultures; Cla_Co, Cladosporium-*Cobetia* co-cultures; Co, *Cobetia* monocultures; Cla_Ps, Cladosporium-*Pseudoalteromonas* co-cultures; Ps, *Pseudoalteromonas* monocultures. Error bars represent standard deviation for three replicates. Different letters indicate statistically significant differences between groups [mean ± SEM, *N* = 3, Van Der Waerden test followed by a *post hoc* test using the Fischer’s least significant difference (LSD), *p* < 0.05].

**FIGURE 7:**
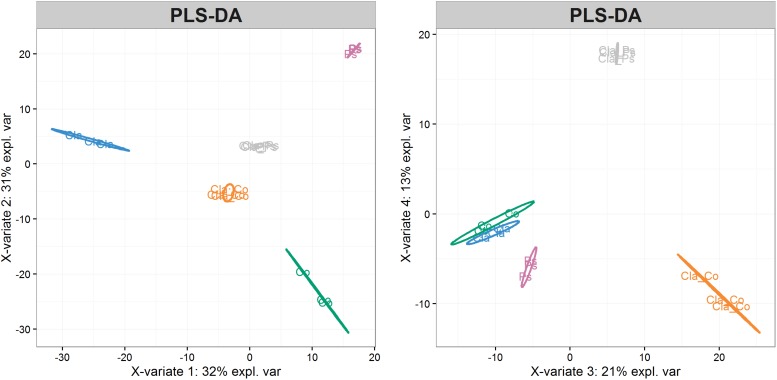
Visualization of samples using the first four latent variables from partial least squares discriminant analysis of 720 selected features. Ps, *Pseudoalteromonas* monocultures (violet); Co, *Cobetia* monocultures (green); Cla, *Cladosporium* monocultures (blue); Cla-Ps, *Cladosporium-Pseudoalteromonas* co-cultures (gray); Cla-Co, *Cladosporium-Cobetia* co-cultures (orange). Ellipses represent 95% confidence intervals.

Metabolites produced in co-cultures of *Cladosporium* SL405T with *Pseudoalteromonas* 352a or *Cobetia* 352b reduced luminescence from *V. campbellii* MM32 by 44 and 20%, respectively. In contrast, metabolites from monocultures of *Cladosporium* SL405T decreased luminescence only slightly (12%), whereas metabolites from monocultures of either bacterium elicited a stronger decrease in luminescence (49%) (Supplementary Data S3). These results highlight the contrasting effects of co-cultures and monocultures on quorum sensing supported by a non-parametric test for independent samples (Van Der Waerden test), followed by a *post hoc* test using the Fischer’s Least Significant Difference (LSD) criterion (Figure 6). Furthermore, the partial least squares discriminant analysis of samples covering the different co-culture conditions characterized through 4,221 metabolomic features collected by LC–MS showed that each monoculture or co-culture is characterized by a specific set of features corresponding to a unique set of metabolites (Supplementary Data S2). Sparse partial least squares discriminant analysis also identified four latent variables of 180 features (720 features in total) that discriminate a culture from all others (Supplementary Data S2). In a second round of partial least squares discriminant analysis based only on these 720 features (Figure 7), the co-cultures were differentiated in the third dimension both from each other and from every monoculture. Analysis of variance of scores get on the 3^rd^ dimension confirmed that selected features significantly separate co-cultures from each other and from monocultures (*F*_2,12_ = 167.2, *p* < 1.7 × 10^–9^). This result implies that bacterial–fungal interactions impact very significantly metabolite production in specific ways.

Features that distinguish a culture and that are altered more than 10-fold over other cultures are listed in Supplementary Table S3. When compared to the corresponding monocultures, 28 and 5 of such features were identified in co-cultures of *Cladosporium* SL405T with *Cobetia* 352b and *Pseudoalteromonas* 352a, respectively. Conversely, 120 and 87 of such features were identified in *Cobetia* 352b and *Pseudoalteromonas* 352a monocultures when compared to the corresponding co-cultures with *Cladosporium* SL405T. Moreover, 93 features diminished by at least a factor of 10 in co-cultures when compared to *Cladosporium* SL405T monocultures. Unfortunately, top-ranked metabolites were not identified by annotation against ISDB (Allard et al., 2016), GNPS, and MassBank. Similarly, identification against SIRIUS 4.0. (Böcker and Rasche, 2008) and Pubchem was inconclusive.

### Search of a Multivariate Link Between Luminescence Measurements and MS Metabolomic Variables

**FIGURE 8:**
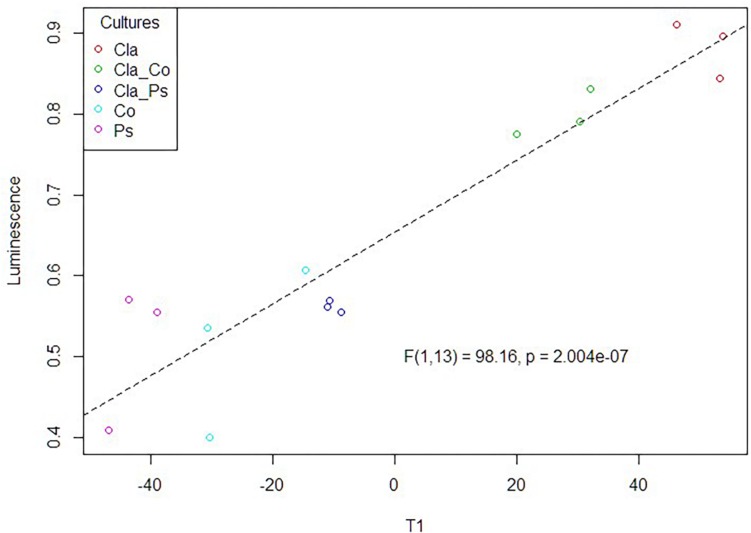
PLS-based regression between the luminescence measurement (QS activity) and the metabolomic profiles obtained in the different culture or co-culture conditions.

**FIGURE 9:**
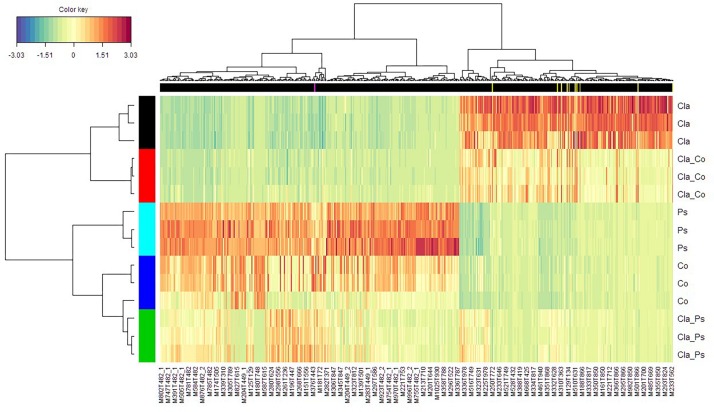
Heatmap displaying both the culture clusters in rows and the clusters of variables (*m/z*) in columns. Ps, *Pseudoalteromonas*; Co, *Cobetia* monocultures; Cla, *Cladosporium* monocultures; Cla-Ps, *Cladosporium-Pseudoalteromonas* co-cultures; Cla-Co, *Cladosporium-Cobetia* co-cultures. For convenience, not all variables are labeled. In the black banner are given variables marked in yellow and pink vertical lines for Cla-Co and Cla-Ps co-cultures, respectively, which were significantly filtered according to (i) a VIP value above 1.20 (and with respective SE values lower than VIP), and (ii) an absolute value of the correlation between the variables selected at the previous step and the PLS component which is above 0.75.

**FIGURE 10:**
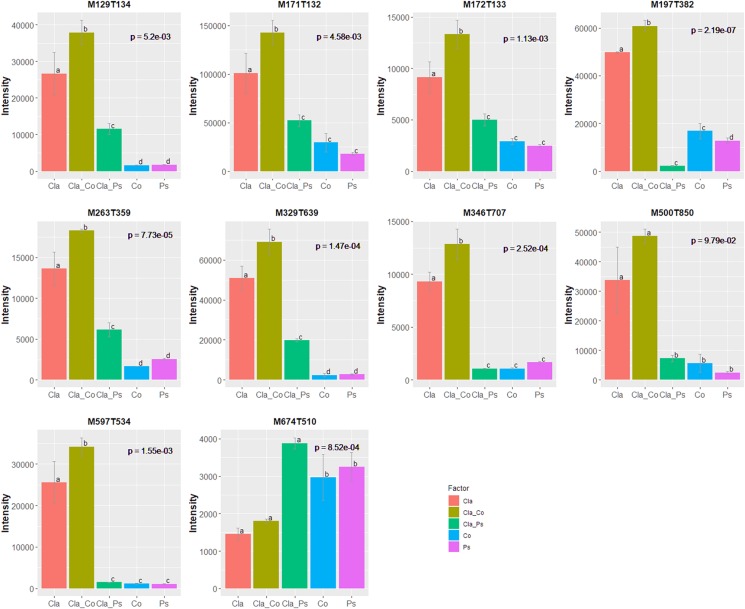
10 variables displaying a correlation with the predicted luminescence response above 0.75 and a higher mean value in co-culture conditions compared to other mono-culture conditions or the alternate co-culture condition. *p*-Values coming from ANOVA are stringently corrected for the false discovery rate when considering the initial set of 4221 variables. Multiple comparisons of means used the Student-Newman-Keuls test.

A link between the global response given by the luminescence representing an integrated measurement of the QS and the metabolome in the different mono or co-culture conditions was obtained thanks a PLS-based regression (Figure 8). Complementary filters such as (i) VIP above 1.20 with a VIP standard error coming from repeated cross-validation calculus lower than the VIP value for the variable, and (ii) absolute value of the correlation between primary metabolomic variables and the predicted luminescence response above 0.75, were used to select from the initial set of 4221 variables a subset of 521 variables. The comprehensive heatmap obtained (Figure 9) showcased nine and one variables displaying a significantly higher mean value for co-culture between Cla and Co and Cla and Ps, respectively (Figure 10). All these 10 significant variables are supposed to be induced in these co-culture conditions when compared to mono-culture conditions as revealed by the multiple comparison of means based on the Student-Newman-Keuls test. Indeed, these variables are of prime importance candidates that would be putatively involved in some metabolic pathways explaining the QS event.

## Discussion

The data highlight the large diversity of cultivable bacterial endophytes associated with healthy *L. digitata*, *S. latissima*, *A. nodosum*, and *P. canaliculata* (Figures 1, 2). The four phyla (*Proteobacteria*, *Firmicutes*, *Actinobacteria*, and *Bacteroidetes*) and 27 genera that were identified are consistent with previous surveys of cultivable bacteria associated with macroalgae (Wiese et al., 2009; Hollants et al., 2013; KleinJan et al., 2017). Notably, the cultivable fraction of bacterial communities appears to vary depending on algal species, sampling site, and algal tissue. Nevertheless, bacteria classified as *Cobetia* and *Pseudoalteromonas* were isolated from every algal species, tissue, and sampling site. Moreover, these genera are the most frequently isolated from *S. latissima*, apart from *Bacillus.* Of note, *Vibrio* and *Flavobacterium* were not isolated from our samples, even though these are frequently isolated from brown algae (Hollants et al., 2013; Albakosh et al., 2016).

Among the isolated bacterial endophytes, 86% were found to produce AI-2, which triggers quorum sensing in the reporter strain *V. campbellii* MM32 (Table 1). *Cobetia, Marinomonas*, and *Erwinia* were the strongest inducers (>100% induction) of quorum sensing, whereas 93 and 73% of isolated *Bacillus* and *Pseudoalteromonas* induced quorum sensing only weakly (<50% induction). These results were confirmed by quantifying DPD, an AI-2 precursor, in bacterial supernatants using tandem mass spectrometry. As shown in Figure 3, *Cobetia* 352b and *Marinomonas* 424 produced around 700 nM of DPD, suggesting that these strains engage neighboring bacteria with AI-2 receptors. *Pseudoalteromonas* 352a also produced DPD but to a lesser extent (66 nM). Interestingly, fungal endophytes isolated along with these bacteria positively or negatively modulated AI-2 quorum sensing (Figures 4, 5). These results provide more evidence that AI-2 quorum sensing is involved in interkingdom signaling.

While inhibitory activity against quorum sensing was previously detected in marine fungi (Martín-Rodríguez et al., 2014), this is the first demonstration, to the best of our knowledge, that fungal metabolites may also enhance quorum sensing. However, such result is not surprising, as this effect was previously observed in metabolites from some other types of eukaryotes such as *Chlamydomonas reinhardtii* and *Chlorella* spp. microalgae (Rolland et al., 2016). Collectively, these findings suggest a key role for AI-2 signaling among endophytes of brown algae. On the other hand, we note that 14 fungal endophytes are strongly antimicrobial against the *Vibrio* biosensor (Figure 5), and thus may be similarly active against *V. harveyi*, a prominent pathogen in aquaculture (Zhang and Li, 2016).

As the fungus *Cladosporium* SL405T and the bacteria *Pseudoalteromonas* 352a and *Cobetia* 352b were isolated from the same holobiont (*S. latissima*), they were characterized in monoculture and in co-culture, with a view to assess the impact of fungal–bacterial interactions on metabolite production and quorum sensing. The data indicate that these isolates produce quorum sensing or quorum sensing-modulating compounds. Of note, these genera are frequently isolated from macroalgae (Hollants et al., 2013; Hulikere et al., 2016; Li et al., 2017). *Pseudoalteromonas* is of particular interest, as it was shown to produce antimicrobials or bioactive molecules against algal spores, invertebrate larvae, fungi, and other bacteria. Such molecules may help the host against surface colonization by these organisms (Holmström et al., 2002; Richards et al., 2017). Also, *Pseudoalteromonas* was implicated in Hole-Rotten disease (Wang et al., 2007).

As shown in Figures 6, 7, co-cultures of Cladosporium SL405T with two different bacteria produce different metabolites. These metabolites are also different from those produced by corresponding monocultures. For example, 28 and 5 metabolic features were at least 10-fold more abundant in co-cultures of *Cladosporium* SL405T with *Cobetia* 352b and *Pseudoalteromonas* 352a than in corresponding monocultures. This result implies that microbial interactions induce production of specific metabolites (Supplementary Table S3). Conversely, 120 and 87 features were at least 10-fold more abundant in *Cobetia* 352b monocultures and *Pseudoalteromonas* 352a monocultures than in co-cultures, suggesting either that production of these metabolites is inhibited by microbial interaction, or that these metabolites are degraded in co-cultures. Similarly, 105 and 108 features (of which 93 are common) were at least 10-fold more abundant in *Cladosporium* SL405T monocultures than in co-cultures with *Cobetia* 352b and *Pseudoalteromonas* 352a.

Taken together, these results demonstrate that metabolomes in co-cultures fundamentally differ from metabolomes in monocultures due to microbial interactions. Unfortunately, chemical characterization of culture-specific metabolites was not possible since none matched known natural products. Identification of the source of metabolites in co-cultures also remains a major challenge, since the structure of such metabolites and other related biochemical information would be required (Xu et al., 2018). Nevertheless, we found that different cultures have variable impact on quorum sensing (Figure 6), such that metabolites obtained from co-cultures of the same fungus with two different bacteria clearly display contrasting effects on quorum sensing (Figure 6). For instance, metabolites from a co-culture of *Cladosporium* SL405T and *Pseudoalteromonas* 352a diminish luminescence from the biosensor by 40%, while metabolites from a co-culture of *Cladosporium* SL405T and *Cobetia* 352b led to a loss of only 20%. The link between the impact on quorum sensing and the metabolites present in different culture conditions was strengthened by the PLS-regression based analysis highlighting 10 variables, highly correlated with the predicted luminescence response (absolute value of the correlation above 0.75), and specially induced in the co-culture conditions when compared to mono-culture conditions (Figure 10).

Collectively, our data provide the first evidence of quorum sensing and quorum quenching in bacterial and fungal endophytes of brown algae. These results highlight the importance of chemical communication among microbial components of a holobiont. Indeed, our laboratory model clearly demonstrates the impact of interspecies interactions on the production of metabolites, including metabolites involved in quorum quenching or in antagonizing other microorganisms. Our model also demonstrates the various phenotypes that may be observed in a given fungal or bacterial strain depending on environmental conditions. Hence, these results provide a glimpse of the complexity of molecular dialogues in the holobiont, and how this may impact host fitness.

Accordingly, the data also highlight the need to fully understand the functional role of all microbial members in the seaweed holobiont and their impact on algal fitness either in nature or in farms. Indeed, quorum sensing is already known to significantly affect the expression of virulence genes in aquaculture pathogens (Zhao et al., 2015). Quorum sensing was also demonstrated to control microbial colonization in the red algae *Delisea pulchra*, notably by release of halogenated furanone, which inhibits pathogenic epiphytic bacteria such as *Nautella* sp. (Harder et al., 2012). Hence, quorum sensing represents a very promising target in future studies of approaches to limit pathogenic effects in algae.

## Data Availability

The datasets generated for this study can be found in GenBank, SUB4670131.

## Author Contributions

AT carried out the experiments. AT, AP, RL, CH, KE, and SP analyzed the data. AT, RL, and SP wrote the manuscript. All authors conceived this study.

## Conflict of Interest Statement

The authors declare that the research was conducted in the absence of any commercial or financial relationships that could be construed as a potential conflict of interest.
