# Supplementary material for: Bacterial–Fungal Interactions in the Kelp Endomicrobiota Drive Autoinducer-2 Quorum Sensing
Source: Front Microbiol. 2019 Jul 31;10:1693. doi: 10.3389/fmicb.2019.01693 (PMC6685064; doi:10.3389/fmicb.2019.01693)
Supplement: Supplementary file 1 [file Data_Sheet_1.docx]

Variation of the luminescence of the biosensor *V.campbellii* MM32 by the bacterial supernatants, calculated as described in the materials and methods section. Error bars represent standard deviation for three replicates.

Variation of the luminescence of the biosensor *V.campbellii* MM32 by the bacterial supernatants, calculated as described in the materials and methods section. Error bars represent standard deviation for three replicates.

Variation of the luminescence of the biosensor *V. campbellii* MM32 by the bacterial supernatants, calculated as described in the materials and methods section. Error bars represent standard deviation for three replicates.

Variation of the luminescence of the biosensor *V.campbellii* MM32 by the bacterial supernatants, calculated as described in the materials and methods section. Error bars represent standard deviation for three replicates.
