# Supplementary material for: Bacterial–Fungal Interactions in the Kelp Endomicrobiota Drive Autoinducer-2 Quorum Sensing
Source: Front Microbiol. 2019 Jul 31;10:1693. doi: 10.3389/fmicb.2019.01693 (PMC6685064; doi:10.3389/fmicb.2019.01693)
Supplement: Supplementary file 2 [file Data_Sheet_2.docx]

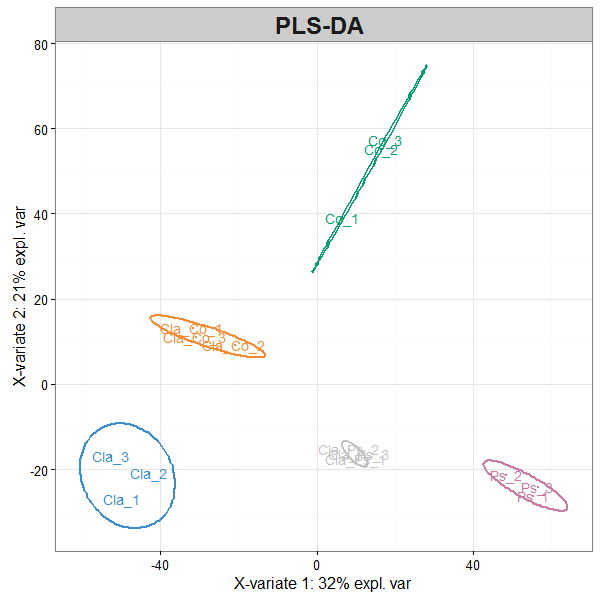


Sample representation using the first 2 latent variables from PLS-DA. Ps: *Pseudoalteromonas* mono-cultures (violet)*,* Co: *Cobetia* mono-cultures (green)*,* Cla: *Cladosporium*  mono-cultures (blue). Cla-Ps: *Cladosporium-Pseudoalteromonas* co-cultures (grey)*,* Cla-Co: *Cladosporium- Cobetia* co-cultures (orange). Ellipses represent a 95% confidence interval.


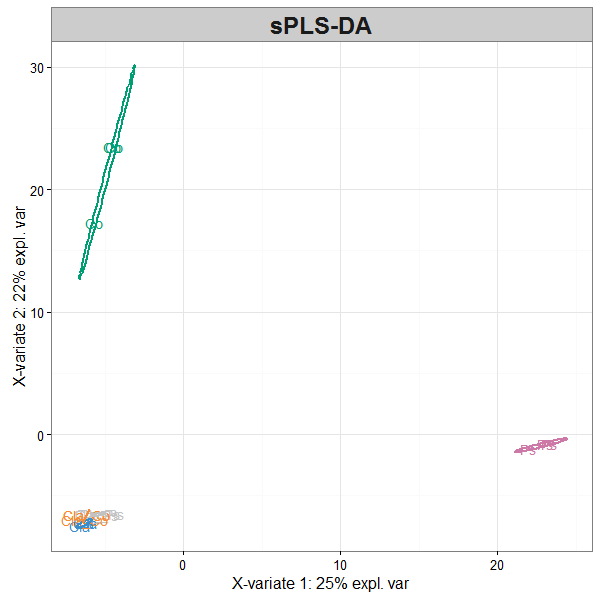

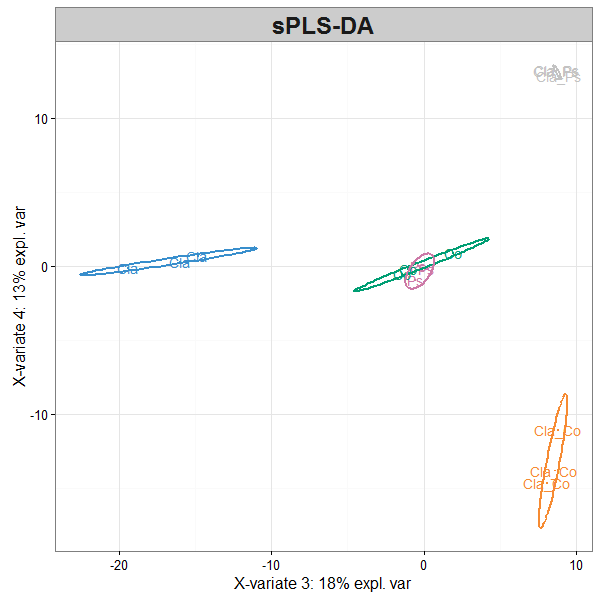


Sample representation using the first 4 latent variables from sPLS-DA (180 features selected on each dimension). Ps: *Pseudoalteromonas* mono-cultures (violet)*,* Co: *Cobetia* mono-cultures (green)*,* Cla: *Cladosporium*  mono-cultures (blue). Cla-Ps: *Cladosporium-Pseudoalteromonas* co-cultures (grey)*,* Cla-Co: *Cladosporium- Cobetia* co-cultures (orange). Ellipses represent a 95% confidence interval.
