# Supplementary material for: Bacterial–Fungal Interactions in the Kelp Endomicrobiota Drive Autoinducer-2 Quorum Sensing
Source: Front Microbiol. 2019 Jul 31;10:1693. doi: 10.3389/fmicb.2019.01693 (PMC6685064; doi:10.3389/fmicb.2019.01693)
Supplement: Supplementary file 3 [file Data_Sheet_3.docx]

SUPPLEMENTARY DATA S3


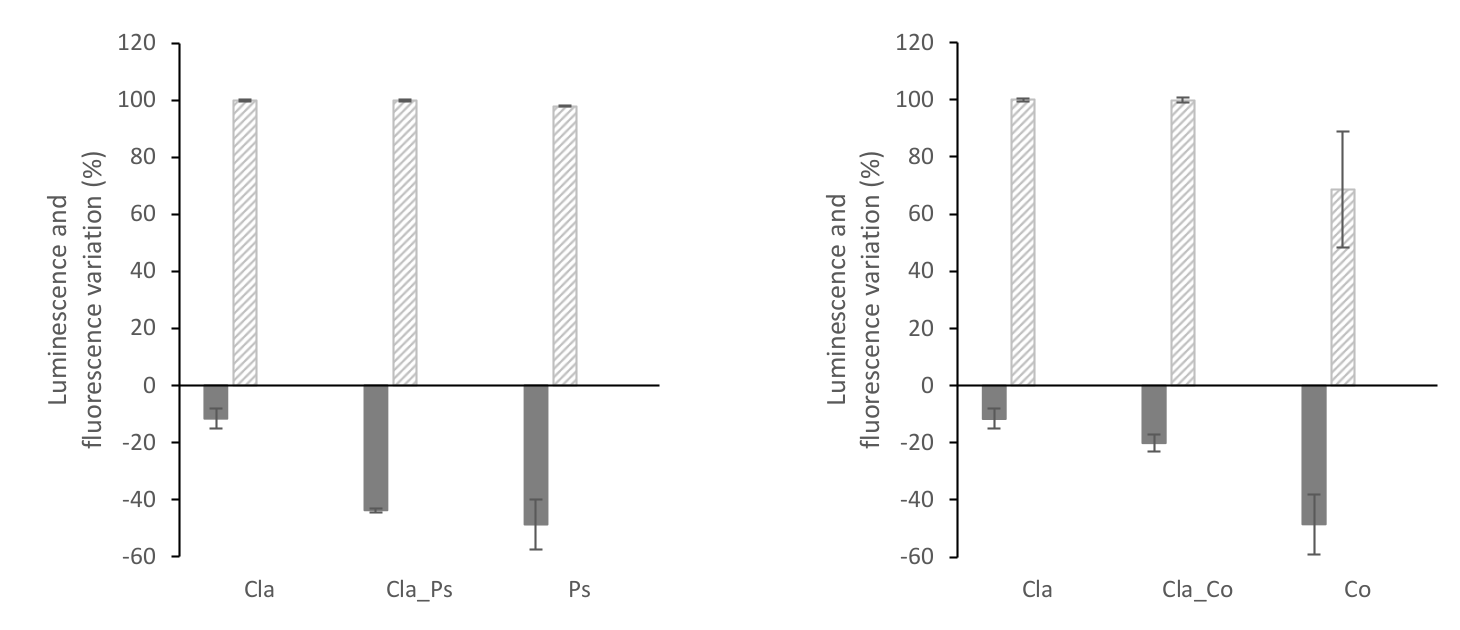


Impact of extracts from mono- and co-cultures on the luminescence in (grey bars) and viability of (hatched bars) the biosensor *V. campbellii* MM32. Cla, *Cladosporium* monocultures; Cla_Co, *Cladosporium-Cobetia* co-cultures; Co, *Cobetia* monocultures; Cla_Ps, *Cladosporium-Pseudoalteromonas* co-cultures; Ps, *Pseudoalteromonas* monocultures. Error bars indicate standard deviation.
