# Supplementary material for: Bacterial–Fungal Interactions in the Kelp Endomicrobiota Drive Autoinducer-2 Quorum Sensing
Source: Front Microbiol. 2019 Jul 31;10:1693. doi: 10.3389/fmicb.2019.01693 (PMC6685064; doi:10.3389/fmicb.2019.01693)
Supplement: Supplementary file 4 [file Table_4.DOCX]

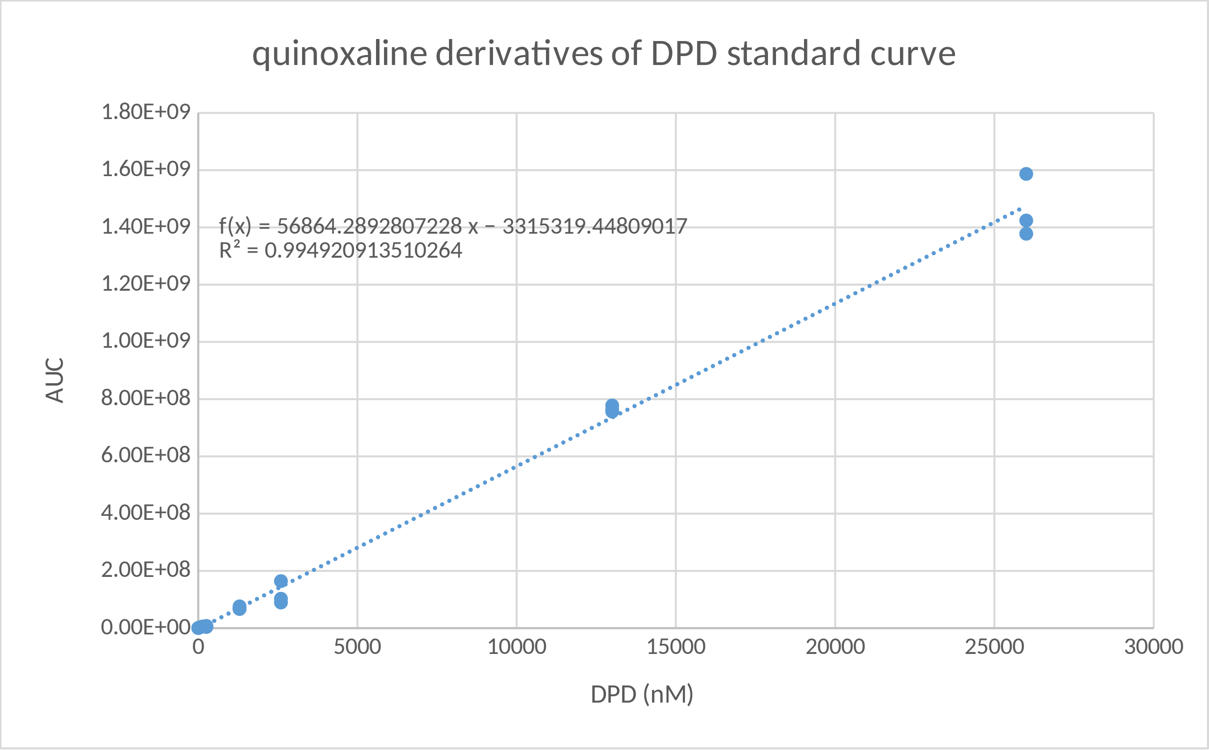
quinoxaline derivatives of DPD standard curve
f(x) = 56864.2892807228 x − 3315319.44809017
R² = 0.994920913510264
AUC
DPD (nM)
1.80E+09
1.60E+09
1.40E+09
1.20E+09
1.00E+09
8.00E+08
6.00E+08
4.00E+08
2.00E+08
0.00E+00
0
5000
10000
15000
20000
25000
30000
